# Use of Statistical and Acoustic Cues for Speech Segmentation in French-Learning 7-Month-Old Infants and French-Speaking Adults

**DOI:** 10.1162/opmi_a_00184

**Published:** 2025-01-23

**Authors:** Mireia Marimon, Elena Berdasco-Muñoz, Barbara Höhle, Thierry Nazzi

**Affiliations:** Center for Brain and Cognition, Universitat Pompeu Fabra, Barcelona, Spain; Integrative Neuroscience and Cognition Center, CNRS - Université Paris Cité, Paris, France; Cognitive Sciences Unit, Department of Linguistics, University of Potsdam, Potsdam, Germany

**Keywords:** Iambic-Trochaic law, transitional probabilities, statistical learning, acoustic cues

## Abstract

Young infants can segment continuous speech with acoustic as well as statistical cues. Understanding how these cues interact can be informative about how infants solve the segmentation problem. This study investigates the use of acoustic and statistical cues by both adult French speakers and 6-to-7-month-old French-learning infants. Both groups were familiarized with a naturally recorded string, alternating either in duration (long-short) or in intensity (soft-loud). In addition, statistical cues were present in both strings, signaling different word boundaries than the acoustic cues. The adults were tested in a recognition task and the infants with the Head-turn Preference Procedure. Results show that the French-speaking adults segmented the strings by responding to the acoustic cues in both familiarization conditions, following the predictions of the Iambic-Trochaic Law. In contrast, the French-learning infants displayed segmentation based on TPs in the Intensity condition only. These findings collectively contribute to our understanding of how the use of acoustic and statistical cues to decode linguistic input changes between infancy and adulthood and differs across languages.

## INTRODUCTION

The speech signal is a continuous stream in which, contrary to many written language systems, words are not separated by blanks. Therefore, a crucial step in language acquisition is to learn to parse the continuous speech stream into possible word form candidates. To solve this segmentation problem, infants are thought to rely on different perceptual and computational mechanisms that exploit properties of the speech signal (Jusczyk, Hohne, & Bauman, 1999; Mattys & Jusczyk, 2001). Experiments with young infants suggest that they can make use of statistical regularities between speech sounds (e.g., Aslin et al., 1998; Saffran et al., 1996) as well as prosodic information (e.g., Jusczyk, Houston, & Newsome, 1999; Nazzi et al., 2006) to solve the segmentation problem. The present study explores the interaction between these two types of cues in French-learning infants and French-speaking adults in a speech segmentation task.

There is evidence that both adults and young infants can track the frequency of co-occurrence of adjacent syllables (transitional probabilities, TPs) (Aslin et al., 1998; Langus et al., 2012; Pelucchi et al., 2009; Saffran et al., 1996; for a review see Krogh et al., 2013). These co-occurrence patterns are informative for word segmentation because the probability of two syllables following each other is higher within a word than between words (Aslin et al., 1998). Although newborns are already sensitive to statistical structure in a continuous artificial speech stream (Fló et al., 2019; Teinonen et al., 2009), it is from 5 months on that English-learning infants have been shown to use TPs for word segmentation (Aslin et al., 1998; Thiessen & Erickson, 2013) also from a natural unfamiliar language (Italian; Pelucchi et al., 2009). This ability has also been found in infants learning other languages like Dutch (Johnson & Tyler, 2010) and French (Hoareau et al., 2019; Mersad & Nazzi, 2012), but only under optimal circumstances, that is, when words are of uniform length. For example, Mersad and Nazzi (2012) found that 8-month-old French-learning infants can segment words of uniform length (trisyllables) from an artificial language string but were only able to segment words of non-uniform length (mixing disyllables and trisyllables) when the string contained the familiar word “maman” (‘mommy’). Statistical learning is seen as a cognitive ability that could help young infants to segment the continuous speech without any previous knowledge about the language that surrounds them, although recent findings suggest it is modulated by the quantity of input previously received (Hoareau et al., 2019).

Another line of research has shown that infants can also segment continuous speech based on prosodic cues, acoustically characterized by changes in pitch, intensity, and duration. Initial research in the framework of the prosodic bootstrapping account has assumed that infants use the predominant stress pattern of their language to segment words from continuous speech (Houston et al., 2000; Jusczyk, Houston, & Newsome, 1999). However, this account has been criticized as not solving “the chicken and egg problem”, since detecting the predominant word stress pattern relies on the previous segmentation of some words of the language (Saffran & Thiessen, 2003). One other line of research has suggested that infants could solve this issue by learning the rhythmic unit of their native language based on a sensitivity to language rhythms already present at birth (e.g., Nazzi et al., 1998), leading to cross-linguistic differences in early segmentation abilities. For instance, French-learning infants rely on syllables (Goyet et al., 2016; Nazzi et al., 2006). An alternative approach to addressing this issue involved suggesting the existence of an innate acoustic bias, the Iambic-Trochaic Law (ITL; Hayes, 1985) that might serve as a bootstrap into word segmentation (Bhatara et al., 2013, 2016; Bion et al., 2011; Hay & Diehl, 2007). The ITL has been proposed as a general perceptual mechanism governed by abstract, universal principles and states that (a) elements contrasting in intensity form groupings with initial stress and (b) elements contrasting in duration form groupings with final stress. Therefore, the predictions of the ITL on the perception of speech and non-speech acoustic stimuli are that listeners will perceive a sequence of *loud-soft-loud-soft* sounds (alternating in intensity) as initial prominence pairs (loud-soft) thus following a strong-weak pattern. In contrast, they will perceive a sequence of *long-short-long-short* sounds (alternating in duration) as final prominence pairs (short-long) thus following a weak-strong pattern. These predictions were confirmed in numerous studies with adults that also establish some cross-linguistic variation. In short, these studies suggest that the ITL effects on perception are modulated by properties of the phonological system of the native language (Abboub, Boll-Avetisyan, et al., 2016; Bion et al., 2011; Hay & Diehl, 2007; Iversen et al., 2008; Nespor et al., 2008). Whether the ITL also guides infants’ early speech segmentation is less clear, especially when considering the impact of cross-linguistic differences. Previous studies found segmentation effects conforming to the ITL in infants between 6 and 9 months of age (Hay & Saffran, 2012; Trainor & Adams, 2000), but the use of cues and their relative balance appears to differ across languages and ages, with potential effects due to differences in the methodology and stimuli used (Abboub, Boll-Avetisyan, et al., 2016; Bion et al., 2011; Molnar et al., 2016; Yoshida et al., 2010).

The current study compares the effects of statistical cues (TPs) and acoustic cues (intensity and duration) on the speech segmentation abilities of French-speaking adults and French-learning infants. More specifically, we investigate whether one type of cue is weighted more heavily in speech segmentation based on the predictions that arise from the ITL, that is, when the effects of intensity and duration are separately tested. Recent studies that focused on the roles of the different cues in word segmentation set up experimental situations in which TPs conflict with prosodic cues without considering the potentially modulating effect of the acoustic correlates of prosodic salience. In adults, results from these studies do not provide a homogeneous picture: While some studies have shown that statistical information is given more weight when both prosodic and statistical cues are present (Mattys et al., 2005), other studies have found that prosodic cues override TPs in word segmentation (Fernandes et al., 2007; Gambell & Yang, 2006; Langus et al., 2012; Marimon et al., 2022, 2024; Vroomen et al., 1998). In infants, when different cues offer conflicting information about word boundaries, language-specific cues were given more weight than TPs in word segmentation in most of the studies (phonotactic cues: Mattys et al., 1999; lexical stress: Johnson & Seidl, 2009; Marimon et al., 2022, 2024; coarticulation cues: Johnson & Jusczyk, 2003; single acoustic cues: Hay & Saffran, 2012). Furthermore, some cross-linguistic variation has been found: While English-learning infants seem to shift from using TPs at 7 months to prosodic cues at 9 months (Thiessen & Saffran, 2003), German-learning infants already rely on prosodic cues at the age of 6 months (Marimon et al., 2024) and later start to consider TPs as an alternative to lexical stress for indicating word boundaries (Marimon et al., 2022). This is probably because the realization and location of lexical stress in words varies across languages, such that it must be acquired through exposure to the native language prosody. Therefore, it is crucial to explore whether and how these cross-linguistic differences affect the relative weight given to the acoustic and statistical cues in the acquisition of different languages. French is an interesting case to study because, unlike English or German, it exhibits no lexical stress. The present study with French-speaking adults and French-learning infants will make use of the procedure to put statistical and acoustic cues in conflict as introduced by Thiessen and Saffran (2003) and extended by Marimon et al. (2022). The present study differs from this previous work in the population tested (7-month-old French-learning infants) and, most importantly, it additionally explores whether the conflict between statistical and prosodic information is acoustically based on intensity or on duration cues, and not on all the prosodic cues indicating stress.

As mentioned above, our interest in French comes from the fact that it does not use contrastive stress at the word level (Cutler & Mehler, 1993; Féry et al., 2011; Goedemans & van der Hulst, 2009). Instead, it has fixed phrasal stress falling on the final syllables of prosodic phrases, which is acoustically realized by increased duration and F0 movement (pitch rise if the phrase is sentence-internal, pitch fall if the phrase is sentence-final; Delattre, 1963; Jun & Fougeron, 2002; Rolland & Loevenbruck, 2002). This has implications for linguistic processing, especially in speech perception. For instance, adult French speakers have been shown to have difficulties in encoding contrastive stress: Although they can discriminate between weak-strong and strong-weak sequences (Michelas et al., 2018; Schwab & Llisterri, 2011), their ability to encode stress under more challenging conditions is limited (Dupoux et al., 1997, 2001, 2008, 2010). Sensitivity to stress in French-learning infants presents similar limitations and modulations by task requirements: While 6-month-olds are able to discriminate between lists of varied strong-weak and weak-strong sequences (Höhle et al., 2009), 9-to-10-month-olds showed this ability only when presented with a single strong-weak and weak-strong sequence (Skoruppa et al., 2009), and required a longer exposure to the stimuli (Bijeljac-Babic et al., 2012).

Despite their low performance in encoding stress information, adult French speakers group syllables in a continuous speech string according to the predictions of the ITL when the syllables alternate in duration or intensity (Bhatara et al., 2013, 2016; Hay & Diehl, 2007) indicating that they can appropriately process these acoustic cues. However, compared to adult German speakers, French speakers respond less consistently and need more salient acoustic cues, suggesting that the prosodic properties of French also affect ITL perception (Bhatara et al., 2013). As for infants, Abboub, Boll-Avetisyan, et al. (2016) tested French- and German-learning 7.5-month-olds in sequences where syllables alternated in pitch (high, low), duration (long, short) and intensity (loud, soft) using the Head-turn Preference Procedure. In the duration and pitch conditions, both German- and French-learning infants looked longer at the items that had been stress-final in the familiarization compared to stress-initial items, suggesting ITL-conform grouping (a familiarity effect for duration, a novelty effect for pitch). There was no significant effect for intensity. In addition, based on the novelty effect found, the authors suggested that pitch cues were easier to use than the other cues. These findings show no cross-linguistic variation and provide supporting evidence for the ITL for the duration and pitch cues. The lack of a significant grouping effect for intensity suggests that both French- and German-learning infants might not be as sensitive to variations in stress patterns based on intensity changes. However, unlike the present study, the stimuli used were synthesized and the prosodic properties artificially controlled. The outcomes of this study will shed more light on the interplay of perceptual mechanisms and language processing, and the role that those play in language acquisition.

The current study addresses for the first time the relative weighting of TPs and of the acoustic cues signaling stress (intensity, pitch, duration) in 6-to-7-month-old French-learning infants and French-speaking adults. Both infants and adults were familiarized with a continuous speech stream in which acoustic cues were pitted against TPs. To do so, we followed a similar design as in Hay and Saffran (2012) by exposing participants to two types of familiarization strings. However, differently from their study, stress was instantiated by different acoustic cues, using either a string alternating in duration (long and short syllables) (Duration Familiarization) or a string alternating in intensity (loud and soft syllables) (Intensity Familiarization). Pitch was not explored as a unique cue because the original formulation of the ITL makes no predictions about pitch (but see Nespor et al., 2008 for a proposal). Following TP information in the Intensity familiarization string would lead to weak-strong groupings (contrary to the ITL prediction), while following TP information in the Duration familiarization string would lead to strong-weak groupings (also contrary to the ITL prediction). In the test phase, disyllabic words were presented that either had occurred with high TPs or had formed a rhythmic unit according to the ITL (weak-strong or strong-weak, depending on the familiarization) in the familiarization string. As a control, additional disyllabic words constructed of syllables that were present but had never occurred adjacently in the familiarization string were also presented during the test phase.

Based on prior research, we could expect two potential outcomes for French adult speakers. Because French adult speakers can segment a speech stream with TPs when no prosodic cues are present (Bonatti et al., 2005; Mersad & Nazzi, 2011), we would expect a segmentation of the words with high TPs (1.0) from the string in both the Duration and the Intensity conditions. However, there is also evidence that French speakers show ITL grouping with a duration cue and with an intensity cue (Bhatara et al., 2013, 2016). Therefore, if they rely more strongly on the acoustic cues of the string, we would expect successful grouping according to the acoustic cues in both conditions (stress-initial for the Intensity Familiarization; stress-final for Duration Familiarization). Regarding the infant group, we could also anticipate two possible results. Drawing insights from studies showing that French-learning infants can segment a string following TPs (Hoareau et al., 2019; Mersad & Nazzi, 2012) and aligning with the principles proposed by Thiessen and Saffran (2003), it is possible that infants would lean toward TPs as a guiding principle for segmentation. Nonetheless, the ITL literature suggests that French-learning infants also have a perceptual bias for prominence grouping when cued by duration or pitch (Abboub, Boll-Avetisyan, et al., 2016, also in newborns, Abboub, Nazzi, & Gervain, 2016). Factoring in what we know of the properties and the acquisition of the French language, where duration is relevant for the phrase level, it is possible that infants might focus predominantly on the duration cue. In that case, we would expect differences between the Intensity and the Duration condition, namely that segmentation in the Intensity familiarization could only be done following TPs, while segmentation in the Duration familiarization could be done following either the statistical or the acoustic cues. Our experimental design including the presentation of non-words in the test phase will allow us to determine which of these cues they will use.

## EXPERIMENT 1: ADULT FRENCH SPEAKERS

### Materials and Methods

#### Participants.

A group of 24 adult monolingually-raised speakers of French were tested (8 males, 16 females, age range: 19–35 years, mean: 25.7). Two additional adults were tested but excluded because of test session interruption (1) or the participant not understanding the task (1). Before the test, participants completed a detailed language background questionnaire. Participants who had been exposed to other languages while growing up were not included in the sample. Participants confirmed that they had no prior history of hearing or speech difficulties. Written informed consent was acquired from all participants. The study received ethical approval from the Ethics Committee of Université Paris Descartes (2011-03).

#### Materials.

##### Familiarization Phase.

We used two familiarization language strings, one where syllables alternated between long and short (Duration Familiarization) and one where syllables alternated between loud and soft (Intensity Familiarization). Both were created from eight syllables (/*bi*/, /*ze*/, /*ko*/, /*my*/, /*zu*/, /*ro*/, /*mu*/, /*gi*/) that were combined into four words (/*bize*/, /*komy*/, /*zuro*/, /*mugi*/). We used similar syllables as Thiessen and Saffran (2003) but adapted them to the French language phonetics. The syllables were phonotactically legal in French and were low in frequency (range token frequency: 0.0-12.49; LEXIQUE; New et al., 2001). None of the disyllabic words or their combinations in the familiarization string formed a real French word.

The syllables were recorded in a sound-attenuated booth by a female monolingual French speaker, trained as a linguist and speech therapist. She was asked to pronounce the stimuli in a lively voice as if she was talking to an infant (mild infant-directed speech). Each syllable was produced with the carrier syllable ‘ke’ in 4 different versions: in a strong and in a weak position within a strong-weak sequence (e.g., strong position: /*’bike*/; weak position: /*’kebi*/) and in a strong and in a weak position within a weak-strong sequence (e.g., strong position: /*ke’bi*/; weak position: /*bi’ke*/). The speaker was able to pronounce the prosodically different sequences following a contrastive stress strategy (e.g., first pronouncing /*bi’ke*/ immediately followed by /*’bike*/). None of the syllables were manipulated, so that the prosodic characteristics of natural speech regarding intensity, duration and pitch specific to French were preserved. The eight syllables used in the familiarization were extracted from these recordings and combined into the four words (/*bize*/, /*komy*/, /*zuro*/, /*mugi*/). Because coarticulation effects are most prominent within syllables and considerably reduced at syllable boundaries (e.g., Rubertus & Noiray, 2018), it was possible to create a continuous familiarization stream by splicing the naturally recorded syllables at zero crossing points with PRAAT (Boersma & Weenink, 2018). A summary of the acoustic properties of the syllables used in the familiarization strings are presented in Table 1 (more detailed tables of the acoustic of the single syllables used in the familiarization are in Appendix). All eight syllables were additionally recorded from the same speaker in isolation without a carrier syllable and with a monotonous voice to avoid stress cues for the test trials (Table 2).

**Table 1.** Acoustic properties of syllables used for the familiarization strings

	**Duration (ms)**		**Intensity mean (dB)**		**Mean F0 (Hz)**	
	**Strong**	**Weak**	**Strong**	**Weak**	**Strong**	**Weak**
**Intensity Familiarization** Syllables from strong-weak productions	358 (*SD* = 28)	359 (*SD* = 40)	**78** (*SD* = 2.7)	**70** (*SD* = 2.8)	282 (*SD* = 13.01)	218 (*SD* = 11.6)
**Duration Familiarization** Syllables from weak-strong productions	**492** (*SD* = 30)	**248** (*SD* = 24)	73 (*SD* = 3.3)	73 (*SD* = 2.6)	261 (*SD* = 13.6)	215 (*SD* = 4.52)

**Table 2.** Acoustic properties of test stimuli

**Syllable**	**Duration (ms)**	**Intensity mean (dB)**	**Mean F0 (Hz)**
ko	322	76	244
my	412	78	235
bi	403	73	222
ze	437	75	225
mu	412	78	235
gi	393	75	224
zu	479	73	227
ro	442	75	228
Average	411 (*SD* = 46.1)	75 (*SD* = 2.01)	230.14 (*SD* = 7.44)

The difference between weak and strong syllables in strong-weak sequences (recorded for the Intensity familiarization string) was mostly indicated by intensity (+8 dB in strong syllables) and F0 (+64 Hz), while for weak-strong sequences (recorded for the Duration familiarization string), the difference was mostly indicated by duration (+245 ms in strong syllables) and F0 (+46 Hz). Note that although we did not intend to manipulate pitch, pitch variations were present in both familiarization strings and also indicated a difference between strong and weak syllables. According to previous results (Bhatara et al., 2013; Bion et al., 2011; Langus et al., 2012), the pitch cue would support a strong-weak grouping. Therefore, pitch would favor the same grouping as intensity in the Intensity familiarization condition, whereas pitch would support a different grouping as the duration cue (strong-weak vs. weak-strong) in the Duration condition. This could lead to stronger effects in the Intensity familiarization string. We further examine this issue in the General Discussion.

The eight syllables were combined to create two familiarization strings: one string using the syllables recorded from weak-strong sequences for the Duration Familiarization and one string using the syllables recorded from the strong-weak sequences for the Intensity Familiarization. In the Duration Familiarization, the duration and pitch alternated such that one syllable was always longer and higher in pitch than the adjacent ones. In the Intensity Familiarization, the intensity and pitch alternated such that one syllable was always louder and higher in pitch than the adjacent ones.

There were no pauses and no coarticulation cues between the single syllables in the string. The strings had slightly different durations (Duration Familiarization: 3 min 23 s; Intensity Familiarization: 3 min 16 s). For the first and last syllables not to be an anchor point for segmentation (Hay & Diehl, 2007; Trainor & Adams, 2000; Woodrow, 1909), all strings started and ended with a 10-second amplitude gradual change performed with Audacity (Audacity Team, 2012): an increase at the beginning of the strings and a decrease at the end. In addition, the first syllable of each string was counterbalanced.

**Figure 1.**
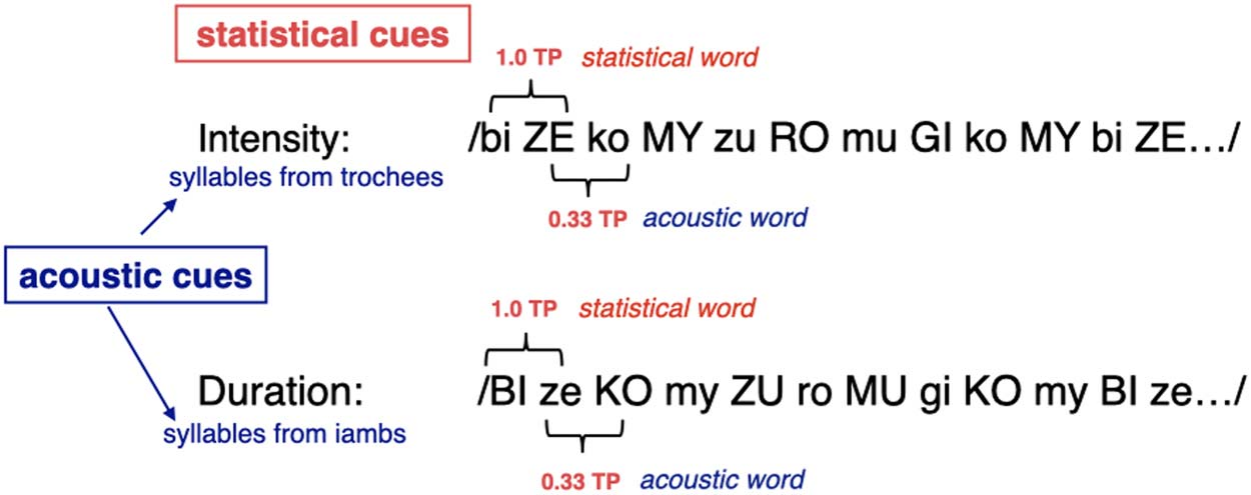
Cues present in the familiarization strings. TP cues are in conflict with the duration and intensity cues in both string. Note that, according to the ITL grouping, pitch properties of the stimuli align with the intensity cues (favoring the strong-weak grouping), but not with the duration cues (disfavoring the weak-strong grouping).

In the familiarization strings, the TPs between syllables conflicted with the acoustic cues for segmenting the string. Following Aslin et al. (1998), we addressed potential disparities in item frequency between acoustic and statistical words in the familiarization stream through the following approach: Two of the statistical words (/*komy*, *bize*/) were presented twice as frequently (90 times each) as the other two statistical words (/*zuro*, *mugi*/), which were each presented 45 times each. As a result, the acoustic words derived from the two more frequently occurring statistical words (/*zeko*, *mybi*/) also appeared 45 times each in the string, matching the frequency of the infrequent statistical words (/*zuro*, *mugi*/). Regarding the statistical cues, the TPs between syllables within the four disyllabic sequences considered as statistical words (/*komy*/, /*bize*/, /*zuro*/, /*mugi*/) were 1.0. The order of presentation of these words in the string was deliberately altered, such that the TPs across the boundaries of these four words were lower than the TPs within the individual words, ranging between 0.2 and 0.4. Immediate repetitions of the same word were not allowed. In terms of acoustic cues, in the Duration Familiarization strings, the first syllable of all the statistical words was consistently strong throughout the string. In the Intensity strings, the second syllable of the statistical words remained consistently strong throughout the string. Figure 1 provides a visual representation of the cues present in the two familiarizations. If participants rely on TPs, they should segment statistical words defined as syllable pairs with a TP of 1.0. In contrast, if participants follow the acoustic cues, they should segment the Intensity string into strong-weak units and the Duration string into weak-strong units. These units—hereafter called acoustic words—straddle the statistical word boundaries.

##### Test Phase.

The stimuli used in the test phase of the experiment consisted of disyllabic words that belonged to three different conditions. These were either the 4 statistical words (the two infrequent statistical words /*zuro*/ and /*mugi*/ and the two frequent statistical words /*bize*/ and /*komy*/), the 4 acoustic words that had the same frequency as the infrequent statistical words (/*zeko*, *mybi*, *giko*, *zemu*/), or four non-words (/*rogi*, *muko*, *zebi*, *zumy*/). The non-words consisted of pairs of syllables that were present but never occurred adjacently during the familiarization (i.e., their TPs were 0.0). The non-word condition was included to serve as a baseline to interpret possible differences in responses between the statistical and acoustic words (Marimon et al., 2022, 2024). The stimuli for the test phase were constructed from the syllables that had been recorded in isolation. Accordingly, they were prosodically flat and participants could not rely on an acoustic match between familiarization and test to identify the words. The acoustic properties of the syllables used to create the test trials are presented in Table 2.

#### Procedure.

The experiment took place in a test booth in front of a computer screen and a keyboard. Prior to the experiment, participants completed a questionnaire about their linguistic background and the consent form. Participants were informed that they would be listening to a string of words for three minutes and would be required to answer questions about these words afterward. They were given the opportunity to seek clarification from the experimenter regarding any instructions. The experiment began when the participant pressed a key to start. During the familiarization phase, the computer screen displayed a gray background with a black loudspeaker icon centered on it. Participants were instructed to listen to the speech and to maintain their focus on the screen throughout the experiment. Following the familiarization, the test phase started, comprising a total of 36 trials, with each word presented three times. The study lasted approximately 7 minutes.

The order of the test trials was randomized for each participant. Each test trial consisted of a single word, played while a loudspeaker icon was displayed on the screen. Following the presentation of each word, participants had to determine whether it had been part of the string they previously heard. They were encouraged to answer promptly and to make their best guess if uncertain. To provide their responses, participants were presented with the words “yes” and “no” positioned on opposite sides of the screen. Participants indicated their choice by pressing one of the two designated keys on the keyboard: the left *Alt* key for “yes” and the right *Alt* key for “no.” The next test trial started either when the participant pressed a key or automatically after 4 s after word-offset. After the test, participants were asked to complete a survey providing feedback about the task. Half of the participants were familiarized with the Intensity string and the other half with the Duration string, but all participants were presented with the same words during the test phase.

#### Analysis.

The outcome measure used in the analysis was the count of ‘yes’ responses recorded for each individual trial, which indicated the participants’ decision that the presented item had been part of the familiarization string. To control for frequency of occurrence in the familiarization string, only the two infrequent statistical words (/*zuro*, *mugi*/) and the two acoustic words formed by the syllables crossing the boundaries of the two frequent statistical words (/*zeko*, *mybi*/) were included in the analysis, along with the four non-words (/*rogi*, *muko*, *zebi*, *zumy*/). Time-out responses, which were instances where participants did not press a response key within 4 s after word-offset, accounted for 0.7% of the total responses and were excluded from the analysis. Statistical analyses were conducted using general linear mixed effects models, employing the *glmer* and *lmer* functions from the *lme4* R package (Bates et al., 2015). Graphs were created using the *ggplot2* package (Wickham, 2009). All data and materials have been made publicly available at OSF and can be accessed at https://osf.io/m87xe/.

First, to compare for differences between conditions, we fit a general linear mixed model to the data in which Test Word Type was entered as a fixed effect with three levels: statistical words, acoustic words and non-words. Condition was coded using a sliding contrast to allow successive comparisons, so that the non-word condition was compared to the two other conditions. The higher number of non-words presented at test was controlled for by adding Test Word Type as a random effect. Familiarization Condition was dummy coded according to the familiarization string, either Duration or Intensity, and was included in interaction with Test Word Type. Participant and Item were included as random effects in the models. The model had the following structure: *glmer*(*yes response* ∼ *Test Word Type* * *Familiarization Condition* + (1|*Participant*) + (1|*Item*) + (1|*Test Word Type*), *family* = *“binomial”*). Secondly, we ran one-sample *t*-tests for each condition and familiarization for comparison to the 50% chance level.

### Results and Discussion

**Figure 2.**
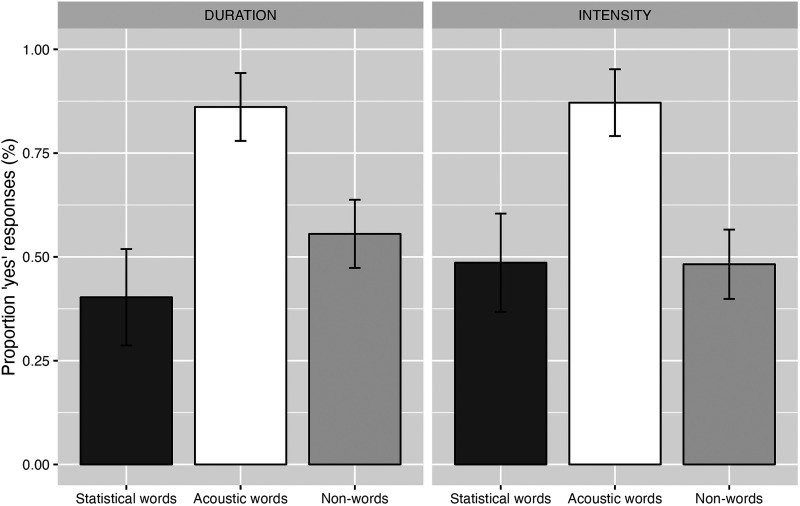
Proportion of correct responses for each familiarization condition (Duration left; Intensity right) for each Test Word Type. Error bars represent the standard error.

Figure 2 shows the proportion of yes-responses by Familiarization Condition and Test Word Type. In the Duration condition, participants responded ‘yes’ to 40.2% of the statistical word trials, to 86.1% of the acoustic word trials and to 55.5% of the non-word trials. In the Intensity condition, participants responded ‘yes’ to 48.6% of the statistical word trials, to 87.1% of the acoustic word trials, and to 48.2% of the non-word trials.

Table 3 presents the complete model output. The model estimated a significant difference between non-words and acoustic words (Nonword − Acoustic, *β* = −1.91, *z* = −3.65, *p* < .001), suggesting that participants gave more ‘yes’ responses for the acoustic words than for the non-words. The difference between non-words and statistical words failed to reach significance (Nonword − Statistical, *β* = −.77, *z* = −1.68, *p* = .094), suggesting that the level of ‘yes’ responses for the non-words and statistical words was similar. Test Word Type did not interact significantly with Familiarization Condition, suggesting that participants behaved the same independently of the familiarization string they heard (i.e., following the acoustic cue in both familiarization conditions). One-sample *t*-tests on the proportion of “yes” responses against chance level (50%) estimated that participants only performed significantly above chance level for the acoustic words for both the Duration and the Intensity strings (see Table 4).

**Table 3.** Output of the fit model for the adult French speakers

*Predictors*	**Affirmative responses**					
	*Estimates*	*Odds Ratios*	*Std. Error*	*CI*	*Statistic*	*p*
(Intercept)	0.66	1.95	0.76	0.90–4.18	1.70	0.088
Test Word Type [Acoustic − Nonwords]	−1.91	0.15	0.08	0.05–0.41	−3.65	**<0.001**
Test Word Type [Statistical − Nonwords]	−0.77	0.46	0.21	0.19–1.14	−1.68	0.094
Familiarization Condition	0.14	1.15	0.60	0.42–3.19	0.27	0.785
Test Word Type [Acoustic − Nonwords]: Familiarization Condition	−0.46	0.63	0.37	0.20–2.00	−0.79	0.432
Test Word Type [Statistical − Nonwords]: Familiarization Condition	0.74	2.11	0.98	0.85–5.25	−1.61	0.107

Observations	571					
Marginal R^2^ / Conditional R^2^	0.238 / A					

**Table 4.** Output of the *t* tests against chance for each familiarization and test word

**Familiarization Condition**	**Test Word Type**	**Estimate**	**Confidence Interval**	***t* Value**	***p* Value**
INTENSITY	Statistical words	0.48	0.236–0.735	−0.12	0.90
	Acoustic words	0.86	0.782–0.956	9.36	**<.001**
	Non-words	0.49	0.307–0.677	−0.09	0.92
DURATION	Statistical words	0.40	0.161–0.643	−0.88	0.39
	Acoustic words	0.86	0.743–0.979	6.73	**<.001**
	Non-words	0.55	0.431–0.679	0.98	0.34

* **Bold** values indicate statistical significance.

In this experiment, we explored which cues adult French speakers favor in the segmentation of a continuous speech string. Our results from the *t*-tests show that, in both familiarization strings (Intensity or Duration), only the proportion of ‘yes’ responses to acoustic words were above chance level, while this was not the case for the non-words and statistical words. In addition, our results from the mixed model show that, in both familiarization strings (Intensity or Duration), acoustic words were more often recognized as having appeared in the string than the non-words, while there was no difference between the statistical words and the non-words. Therefore, it seems that French speakers rely more strongly on acoustic cues compared to TPs when segmenting a continuous syllable string when the two types of cues are in conflict and indicate different word boundaries. Interestingly, French speakers made use of the acoustic cues present in the string, suggesting that duration, as well as intensity, can override TPs in word segmentation. These results contribute further evidence for the perception mechanisms proposed by the ITL (Hayes, 1985), demonstrating that, for French listeners, a string alternating in duration (long, short) will be grouped as weak-strong and that a string alternating in intensity (loud, soft) will be grouped as strong-weak.

Our data from Experiment 1 shows that French adult speakers use acoustic cues for word segmentation. We now explore the comparison between these adult findings and the mechanisms available to infant learners, for whom word segmentation is a critical aspect of native language acquisition. In Experiment 2, we adapted the paradigm to suit the developmental capabilities of infants and we explore the early mechanisms involved in word segmentation in 6-to-7-month-old French-learning infants. Crucially, the preferential use of for acoustic cues in adults does not necessarily imply that infants will exhibit the same behavior. While previous research suggests that grouping preferences as predicted by the ITL emerge between 6 and 9 months and are partly modulated by linguistic experience (Abboub, Boll-Avetisyan, et al., 2016; Bion et al., 2011; Hay & Saffran, 2012; Iversen et al., 2008; Yoshida et al., 2010), and that ITL-based effects are found for duration and pitch but not intensity in French-learning 7.5-month-olds (Abboub, Boll-Avetisyan, et al., 2016), whether the ITL also guides infants’ early speech segmentation is still an open question. To test this, infants were familiarized with the same type of string as used in Experiment 1 in which the statistical and acoustic cues led to different segmentation outcomes and presented with the same types of words at test: acoustic words, statistical words and non-words. The non-words served as a reference point because they have never heard these sequences. Segmentation of either the statistical words or the acoustic words would be attested if orientation times to these words are significantly different from the non-word condition (while a null result is indicative of a lack of segmentation). Given that previous similar studies found novelty preferences (e.g., Black & Bergmann, 2017; Johnson & Tyler, 2010; Marimon et al., 2024), we expected longer orientation times to the non-words than to either the statistical or the acoustic words, indicating which of the two cues more heavily guided infants’ segmentation.

## EXPERIMENT 2: FRENCH-LEARNING INFANTS

### Materials and Methods

#### Participants.

Thirty-two 6-to-7-month-old French-learning infants were tested in Paris, France (9 girls, mean = 7.03; range = 6.16–7.23). Thirty additional infants were tested but excluded due to fussiness (2), crying (20), not looking during more than three trials (4), not looking to the left lamp (1), technical problems (2) and parental intervention (1). All infants were born full-term and did not exhibit apparent health issues. Sample sizes were based on previous studies with a similar design (Bion et al., 2011; Thiessen & Saffran, 2003, *N* = 30, divided into trochaic and iambic condition; Johnson & Jusczyk, 2003, *N* = 16). Please see the General Discussion for a discussion about this sample size. We obtained written informed consent from all families that participated in the study. This study received approval by the Ethics Committee of Université Paris Descartes (2011-03).

#### Materials.

The familiarization strings were the same as in Experiment 1. However, there were two notable differences in the stimuli used for the test trials. First, the number of words presented in each condition was reduced to two: specifically, the two infrequent statistical words (/*zuro*/ and /*mugi*/), the two acoustic words with comparable frequency to the infrequent statistical words (/*zeko*/ and /*mybi*/), and two non-words (/*rogi*/ and /*muko*/). Second, for each of these 6 words, we generated a sound file consisting of 14 repetitions of the word, resulting in a total duration of 18 s for each sound file.

#### Procedure.

We used the Head-turn Preference Procedure, originally introduced by Hirsh-Pasek et al. (1987). During the experimental session, infants were seated on a caregiver’s lap in a sound-attenuated test booth. Both the experimenter and the caregiver wore headphones and listened to music to avoid influencing infant’s behavior. Additionally, the caregiver was explicitly instructed not to intervene or interact with the infant during the experiment. The experimenter controlled the presentation of the stimuli and the activation of the lights based on the infants’ head movements by using the computer mouse. Inside the booth, three lights were securely positioned: a central green light and a red light on each side. Outside the test booth, two loudspeakers were placed just below the red lights. The experimenter remained seated outside the booth and monitored the infants through a camera. The procedure during the familiarization proceeded as follows: Initially, the green light began blinking to capture infants’ attention to the center. Once the infant oriented toward the green light, the experimenter pressed the mouse, causing one of the side lights (randomly selected) to start flashing. The side light continued to flash until the infant looked away for two consecutive seconds. Then the light extinguished and the center light resumed blinking. During familiarization, the speech string was played continuously and was not contingent on infants’ looking behavior to avoid uncontrolled breaks in the familiarization strings. The purpose of incorporating the lights during this phase was to maintain infants’ attention and to familiarize them with the changing positions of the lights. Orientation times (OTs) during the familiarization string were not measured.

Following the completion of the familiarization phase, the test phase started. During the test phase, the presentation of the auditory stimuli was contingent on infants’ looking behavior. Each trial started with the green center light blinking to capture infants’ attention toward the center. Once the infant oriented to the green center light, it ceased blinking and one of the side red lights began to blink. When the infant turned her head toward the blinking side light, the speech stimulus was initiated and played until completion (18 s) or until the infant looked away from the target side for more than two consecutive seconds. If the infant briefly turned her head for less than two seconds, the presentation of the speech file continued, but the time spent looking away was not included in the total OT. Information regarding the duration of OTs was coded by an experimenter using the mouse. The coding experimenter was seated outside the sound-attenuated booth, ensuring that they remained blind to the experimental condition being presented. The test phase consisted of a total of 12 trials. Each word was presented twice, in two different blocks of 6 trials, and each type of stimuli was presented once one the right side and once on the left side in each block.

Half of the infants (*n* = 16) was familiarized with the Duration familiarization string and the other half was familiarized with the Intensity familiarization string. At test, all infants were presented with the same 6 words, each presented twice in two separate blocks (for a total of 12 test trials). In each block, the two instances of each word type were presented on different sides of the booth. There were four different versions of the experiment which differed in the order of stimulus presentation: the blocks differed in the order of presentation the 6 test words (2 statistical, 2 acoustic, 2 non-words). Between infants, the order of presentation of these blocks was counterbalanced. The experimental session lasted between 3 and 5 minutes.

#### Analysis.

The analyses were performed in R using the same packages as in Experiment 1. Trials below 1 s were excluded from the analysis (26 trials from the total observations, 6.78%) because infants would have only heard the first word of the trial. We employed a linear mixed-effects model with raw OTs as the dependent outcome variable. In the model, Test Word Type was entered as a fixed effect with three levels: statistical word, acoustic word, and non-word. As in the adult analysis, Condition was coded using a sliding contrast for successive comparisons between the conditions, taking the non-word word type as the reference. Familiarization Condition, coded according to the two familiarization strings (Duration and Intensity) was coded as a sum-to-zero contrast and included as a between-participant nesting factor in interaction with Test Word Type. We opted for a nested design given that, although we consider that our sample size is large enough to observe main effects in each condition, it is unlikely that we have enough statistical power to observe an interaction between Familiarization Condition and Test Word Type. Age in days (centered) and Block (1 or 2) were included in the model as fixed factors. The model contained random intercepts by-subject (Participant) to allow for differences in baseline OTs. The model had the following structure: *lmer*(*OT* ∼ *Familiarization Condition* / (*Test Word Type* * *Familiarization Condition*) + *Block* + *Age* + (1|*Participant*)^1^.

### Results and Discussion

**Figure 3.**
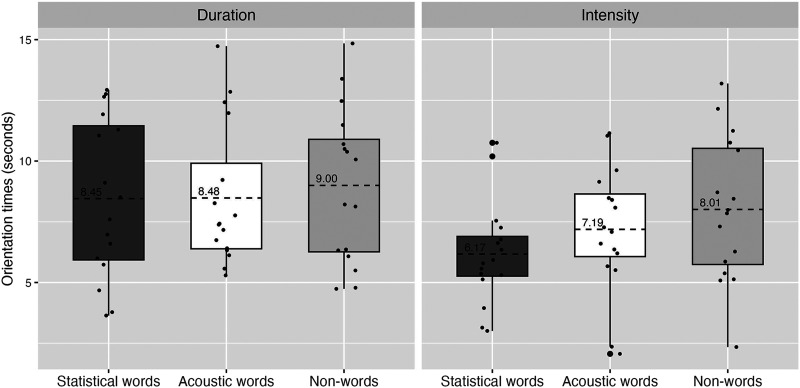
Orientation times for each test word type by familiarization in infants. The dashed line represents the mean. The error bars represent the interquartile range.

The individual and mean raw OTs for each test type by Familiarization are presented in Figure 3. In the Duration familiarization condition, infants listened to the statistical words for 8.45 s (*SD* = 4.66) on average during the test trials, to the acoustic words for 8.48 s (*SD* = 4.51), and to the non-words for 8.99 s (*SD* = 4.98). In the Intensity familiarization condition, infants listened to the statistical words for 6.17 (*SD* = 4.14), to the acoustic words for 7.19 s (*SD* = 4.67), and to the non-words for 8.01 s (*SD* = 4.82) on average during the test trials.

Table 5 presents the complete model output. In the Intensity Familiarization Condition, the results indicate a significant difference between non-words and statistical words at test (*β* = −1883, *t* = −2.46, *p* = .014), while the difference between non-words and acoustic words failed to reach significance (*β* = 679, *t* = 0.87, *p* = .38). In the Duration Familiarization condition, both differences failed to reach significance (Nonword − Statistics: *β* = −585, *t* = −0.76, *p* = .44; Nonword − Acoustic, *β* = 677, *t* = 0.87, *p* = .38). The model also estimated a main effect of Familiarization condition (*β* = −1813, *t* = −2.31, *p* = .022), indicating that infants had overall longer OTs at test when they were familiarized with the Duration string compared to Intensity familiarization string. No other effects reached significance.

**Table 5.** Output of the model for the French-learning infants

*Predictors*	**Orientation time (ms)**				
	*Estimates*	*Std. Error*	*CI*	*Statistic*	*p*
(Intercept)	7855.85	438.87	6992.66–8719.04	17.90	**<0.001**
Familiarization Condition	−1813.54	785.24	−3357.98 – −269.11	−2.31	**0.022**
Block 2-1	113.14	445.81	−763.69–989.97	0.25	0.800
Age (days)-Centered	71.11	37.77	−3.18–145.39	1.88	0.061
Familiarization Duration: Test Word Type Nonword-Acoustic	677.12	776.67	−850.45–2204.68	0.87	0.384
Familiarization Intensity: Test Word Type Nonword-Acoustic	679.28	777.00	−848.94–2207.49	0.87	0.383
Familiarization Duration: Test Word Type Statistical-Nonword	−585.55	773.35	−2106.59–935.49	−0.76	0.449
Familiarization Intensity: Test Word Type Statistical-Nonword	−1883.29	765.88	−3389.65 – −376.94	−2.46	**0.014**

Observations	357				
Marginal R^2^ / Conditional R^2^	0.062 / 0.199				

* **Bold** values indicate that the effect was statistically significant.

Experiment 2 explored which cues 6-to-7-month-old French-learning infants favor in the segmentation of a continuous speech string, pitting acoustic cues against statistical cues. Our results suggest different effects for the two types of acoustic cues (duration or intensity cues) in the familiarization. In the Intensity condition, infants listened significantly longer to the non-words compared to the statistical words, but not compared to the acoustic words. In the Duration condition, no significant difference was found between OTs to the non-words compared to the other two kind of words, suggesting that either they were using neither cue (duration/TPs), or both cues to the same extent. In addition, infants in our sample looked overall significantly longer across the test phase after being familiarized with the Duration condition compared to the Intensity condition.

## GENERAL DISCUSSION

The aim of this study was to investigate the use of acoustic and statistical cues to segment continuous speech in French adult speakers and 6-to-7-month-old French-learning infants. Both adults and infants were familiarized with a language string from naturally recorded syllables, in which acoustic cues (alternation in either duration or intensity cues) indicated different word boundaries than TPs. Adults were then tested in a recognition task (2AFC) and infants were tested with the HPP. Notably, we added a third condition at test (non-word condition) in both experiments, as in Marimon et al. (2022, 2024), to help us interpret the test preference in both adults and infants. Overall, our findings suggest that French adults successfully segmented the strings following the acoustic cues in both familiarization conditions. In contrast, French-learning infants segmented the words from the string following TPs, but only in the Intensity familiarization condition.

Our results from adult French adult speakers are line with research showing that they group syllables in a continuous speech string according to the predictions of the ITL (Bhatara et al., 2013, 2016; Hay & Diehl, 2007), indicating that they can appropriately process these acoustic cues. Crucially, in our study, this finding was found even though TPs were present in the string and indicated different word boundaries than the acoustic cues. To our knowledge, this is the first study investigating cue-weighting in speech segmentation by French adult speakers using naturally recorded stimuli (and therefore natural acoustic cues signaling stress). Although there is plenty of evidence that adults are capable of segmenting speech based on TPs (for a review, see Krogh et al., 2013, or Saffran & Kirkham, 2018), including French (Bonatti et al., 2005; Mersad & Nazzi, 2011), our findings are in line with research indicating that statistical cues seem to be easily overridden by acoustic cues in adults (Fernandes et al., 2007; Langus et al., 2012; Shukla et al., 2007). Besides, since response levels for the non-words were similar to those for the statistical words (no significant difference), our results support the idea that acoustic cues may be the only source of information that participants are using for segmentation.

However, it is possible that this finding is partly related to task characteristics, and that our participants would have needed more exposure time for segmenting words using TPs than the 3 minutes they had in the present study. It is likely that longer exposure could have increased the weight of the statistical cues to the point of reaching segmentation with TPs while dismissing single acoustic cues. However, some studies (e.g., Mersad & Nazzi, 2011) have demonstrated segmentation based on TPs with also a short amount of time (3 min).

This increased weight given to acoustic cues over statistical cues could be accounted for by several explanations. First, as Shukla et al. (2007) suggested, prosodic information seems to suppress the access of statistical information in the auditory domain. Second, acoustic cues are readily available from the first syllables heard, which may benefit a segmentation of the string based on acoustic cues. This is not the case for TPs, as participants need to hear enough syllables to be able to establish the statistical regularities between syllables and use them to segment words. Lastly, learning regularities from the input might be less effective when attention is diverted away from the relevant statistical information, as was the case here given the prosodic variations present in the signal. As Toro et al. (2009) suggested, it is likely that the salient variations in pitch, duration and intensity disrupted the extraction of the statistical information regarding word boundaries and may have had an influence in the segmentation of the string. Nevertheless, the TPs in our stimuli were sufficiently reliable for adults to use for segmentation because they were perfect (1.0 within-words) and less complex compared to those found in natural languages.

Note that a novel aspect from the current study was the use of naturally recorded syllables to create the familiarization string. We aimed to enhance the ecological validity regarding the presented prosodic information, in contrast to the synthesized speech typically utilized in prior research (e.g., Aslin et al., 1998; Thiessen & Saffran, 2003, but see Hay & Saffran, 2012). As a consequence, and although we only meant to manipulate duration and intensity cues, pitch changes were present in our naturally recorded syllables, and differentiated stressed from unstressed syllables in both familiarization strings. Previous research in adults showed that pitch alone leads to the same outcomes on ITL grouping as intensity, i.e., strong-weak grouping (Bion et al., 2011; but not in French adults for the pitch cue, Bhatara et al., 2013, 2016). Therefore, according to the ITL principles, the potential grouping elicited by pitch converged with the intensity cue (Intensity familiarization) but was incongruent with the duration cue (Duration familiarization) in our familiarization strings. If pitch would have influenced the results, it would have facilitated segmentation of acoustic words in the Intensity familiarization condition but would have made segmentation more difficult in the Duration condition. However, French adult speakers in our sample exhibited no difference in segmentation between the two familiarization conditions. Hence, one possibility is that the duration and intensity cues in our study have the same strength, and that pitch was not used to segment (which is consistent with the finding of non-grouping of pitch-varied sequences by adult French speakers, Bhatara et al., 2013). An alternative hypothesis is that the lack of difference between the two familiarization conditions is the result of cue interaction. Indeed, duration could be a stronger word boundary cue than intensity in French, given that the end of phonological phrases is marked with increased duration and F0 movement (pitch rise for sentence-internal; pitch fall for sentence-final, Delattre, 1963; Jun & Fougeron, 2002; Rolland & Loevenbruck, 2002). However, this advantage of duration over intensity would have been cancelled here by the fact that the pitch cue was incongruent with the duration cue but congruent with the intensity cue. Further research will be needed to choose between these two alternatives.

Shifting now our focus to the outcomes related to infants, it becomes apparent that, when presented with identical speech strings for an equivalent duration of familiarization as the adults, infants exhibited a stronger reliance on TPs but only in the Intensity Familiarization condition. Our results suggest different effects for the two types of acoustic cues (duration or intensity cues) present in the familiarization. In the Intensity condition, infants preferred non-words over statistical words, but not over acoustic words, which suggests a use of statistical cues for segmentation. In the Duration condition, there was no clear preference, suggesting infants might have either used both cues (TPs/duration) simultaneously and with equal weight, or none of them. While both options would lead to an apparent segmentation failure, the former explanation is more likely given that infants were using statistical cues in the Intensity condition. This differential effect between the two acoustic cues would be in line with the findings that French-learning 7.5-month-olds use duration but not intensity as a cue to the ITL (Abboub, Boll-Avetisyan, et al., 2016). Therefore, assuming that in the Duration condition infants were using both cues, two outcomes could have been observed: segmentation of both the acoustic and the statistical words, or overall lack of segmentation of both due to interference. The fact that we did not observe a difference in the way infants responded to the non-words compared to both the statistical and prosodic cues suggests an overall lack of segmentation.

Our infant findings are in line with previous studies showing TP use by infants at a young age in different languages (Aslin et al., 1998; Hoareau et al., 2019; Johnson & Tyler, 2010; Mersad & Nazzi, 2012; Pelucchi et al., 2009; Saffran et al., 1996; Thiessen & Saffran, 2003). However, this is the first study that shows segmentation based on TPs by French-learning infants at this young age, around 7 months, hence about 1.5 months younger than in previous studies (Hoareau et al., 2019; Mersad & Nazzi, 2012). Besides, whereas in previous studies no other cues were present, we extended these findings to a cue-weighting situation in which TPs were pitted against acoustic cues, as in Thiessen and Saffran (2003). In such a paradigm, English-learning infants relied more strongly on TPs at the ages of 5 and 7 months (e.g., Thiessen & Erickson, 2013; Thiessen & Saffran, 2003), but changed their reliance to prosodic cues by the ages of 8, 9, and 11 months (Johnson & Jusczyk, 2003; Johnson & Seidl, 2009; Thiessen & Saffran, 2003), establishing that a developmental shift seems to occur for English-learning infants between the ages of 7 and 9 months. A different developmental pattern was found for German, as German-learning infants already relied more strongly on prosodic cues at 6 months (Marimon et al., 2024). For our French-learning 7-month-olds, our results suggest higher reliance on TPs when pitted against intensity, although a shift might have started to happen compared to duration, as the lack of effects in that condition is compatible with an equal reliance on statistical and duration cues. Further investigation will be needed to clarify this point.

Regarding the use of acoustic cues along the principles of the ITL, infants showed no evidence of using either duration or intensity (or pitch) for segmentation. We had predicted based on Abboub, Boll-Avetisyan, et al. (2016) and Abboub, Nazzi, and Gervain (2016) that infants might be able to use the duration cue more effectively than the intensity cues. The fact that only the presence of the duration cue (but not the intensity cue) is blocking the observed effect of the statistical cues could be taken as evidence that, at 7 months, French-learning infants are giving equal weight to statistical and duration cues, and lower weight to the intensity cue. If that is the case, our findings would be in line with Abboub, Boll-Avetisyan, et al. (2016), who showed that French-learning 7.5-month-olds perceive a rhythmic structure when it was cued by duration or pitch but not intensity (for how pitch cues might have affected segmentation performance see previous discussion of adult results).

A final point we would like to address is the sample size and the achieved power in the current study. We employed sample sizes comparable to previous research in related infant language learning studies (e.g., Bion et al., 2011; Hay & Saffran, 2012; Johnson & Jusczyk, 2003; Yoshida et al., 2010), which commonly test 16–22 infants per condition. These sample sizes reflect the challenges inherent in infant research, including recruitment and data collection constraints. However, retrospective power analyses of these previous studies reveal that the achieved power for detecting small to medium effects is often below the conventional threshold of 80% (e.g., ∼27% power for small effects, with 16 infants per condition), calling for cautious interpretation, although medium to large effects typically achieve higher power (∼70–80%). For our study, using G-power, estimated power was of about 70% for the statistical words versus non-words comparison in the intensity condition, close to the conventional 80% threshold. In contrast, the power for the other 3 comparisons was around 10 to 13%, and these findings should be interpreted with caution. To help plan future studies, our dataset is available at https://osf.io/m87xe/.

In conclusion, our findings suggest that French adult speakers successfully segmented the speech strings primarily based on acoustic cues, aligning with the ITL. Interestingly, this occurred even when TPs signaled different word boundaries than the acoustic cues. Conversely, French-learning infants at the age of 7 months gave more weight to TPs during segmentation when it was pitted against intensity, but not when it was pitted against duration. This research contributes valuable insights into cue weighting during language segmentation and speech perception in both infants and adults, establishing a shift between the weighting used in early development and later in adulthood. It emphasizes the need for further investigation for crosslinguistic and age-related factors on these processes.

## ACKNOWLEDGMENTS

We would like to thank T.F., M.D.S., and A.P. for their assistance in setting up the experiment and recruitment. We also thank the BabyLab Team in Paris and all the families that participated in the study. We thank M.H. for the stimuli recording.

## FUNDING INFORMATION

This work was funded by the European Union’s Horizon 2020 research and innovation programme under grant agreement No. 641858 and LABEX EFL grant (ANR-10-LABX-0083).

## AUTHOR CONTRIBUTIONS

M.M.: Data curation; Formal analysis; Funding acquisition; Investigation; Methodology; Project administration; Validation; Visualization; Writing – original draft; Writing – review & editing. E.B.: Investigation. B.H.: Conceptualization; Funding acquisition; Resources; Supervision; Writing – review & editing. T.N.: Conceptualization; Funding acquisition; Methodology; Project administration; Resources; Supervision; Writing – review & editing.

## DATA AVAILABILITY STATEMENT

All data and materials have been made publicly available at OSF and can be accessed at https://osf.io/m87xe/.

## Note

^1^ In response to the question of a reviewer, we explored whether sex affects performance. When adding sex to the model; no significant effect was found (*p* = .41), and the model was not improved, while the other effects were replicated. This aligns with findings from a meta-analysis (Gemignani & Gervain, 2024) showing no effect of sex in statistical learning tasks.

## APPENDIX: DETAILED ACOUSTIC PROPERTIES OF THE FAMILIARIZATION SYLLABLES

### Duration Familiarization Condition

**Table:**

**Syllable**	**Stress**	**Duration (ms)**	**Intensity mean (dB)**	**Mean F0 (Hz)**	**Pitch Peak (Hz)**
ko	weak	448	77	278	320
bi	weak	503	70	250	296
mu	weak	505	75	250	282
zu	weak	514	71	266	299
Average		492 (*SD* = 30)	73.4 (*SD* = 3.3)	261 (*SD* = 13.6)	299 (*SD* = 15.8)
my	strong	264	76	220	230
ze	strong	273	71	217	235
gi	strong	234	70	213	232
ro	strong	222	73	210	223
Average		248 (*SD* = 24)	72.7 (*SD* = 2.6)	215 (*SD* = 4.52)	230 (*SD* = 4.9)

### Intensity Familiarization Condition

**Table:**

**Syllable**	**Stress**	**Duration (ms)**	**Intensity mean (dB)**	**Mean F0 (Hz)**	**Pitch Peak (Hz)**
ko	strong	332	72	210	236
bi	strong	380	69	212	239
mu	strong	387	73	235	276
zu	strong	338	67	215	239
Average		358 (*SD* = 28)	70.4 (*SD* = 2.8)	218 (*SD* = 11.6)	248 (*SD* = 18.72)
my	weak	338	78	291	322
ze	weak	410	76	296	316
gi	weak	369	76	272	326
ro	weak	317	82	270	322
Average		359 (*SD* = 40)	78 (*SD* = 2.7)	282 (*SD* = 13.01)	322 (*SD* = 4.03)
